# Post-Zygotic and Inter-Individual Structural Genetic Variation in a Presumptive Enhancer Element of the Locus between the *IL10Rβ* and *IFNAR1* Genes

**DOI:** 10.1371/journal.pone.0067752

**Published:** 2013-09-04

**Authors:** Hamid Reza Razzaghian, Lars A. Forsberg, Kancherla Reddy Prakash, Szymon Przerada, Hanna Paprocka, Anna Zywicka, Maxwell P. Westerman, Nancy L. Pedersen, Terrance P. O'Hanlon, Lisa G. Rider, Frederick W. Miller, Ewa Srutek, Michal Jankowski, Wojciech Zegarski, Arkadiusz Piotrowski, Devin Absher, Jan P. Dumanski

**Affiliations:** 1 Department of Immunology, Genetics and Pathology, Uppsala University, Uppsala, Sweden; 2 Hematology Research, Mount Sinai Hospital Medical Center, Chicago, Illinois, United States of America; 3 Department of Medical Epidemiology and Biostatistics, Karolinska Institutet, Stockholm, Sweden; 4 Environmental Autoimmunity Group, National Institute of Environmental Health Sciences, National Institutes of Health Clinical Research Center, Bethesda, Maryland, United States of America; 5 Surgical Oncology Clinic, Collegium Medicum, Oncology Center, Nicolaus Copernicus University, Bydgoszcz, Poland; 6 Department of Biology and Pharmaceutical Botany, Medical University of Gdansk, Gdansk, Poland; 7 HudsonAlpha Institute for Biotechnology, Huntsville, Alabama, United States of America; Karolinska Institutet, Sweden

## Abstract

Although historically considered as junk-DNA, tandemly repeated sequence motifs can affect human phenotype. For example, variable number tandem repeats (VNTR) with embedded enhancers have been shown to regulate gene transcription. The post-zygotic variation is the presence of genetically distinct populations of cells in an individual derived from a single zygote, and this is an understudied aspect of genome biology. We report somatically variable VNTR with sequence properties of an enhancer, located upstream of *IFNAR1*. Initially, SNP genotyping of 63 monozygotic twin pairs and multiple tissues from 21 breast cancer patients suggested a frequent post-zygotic mosaicism. The VNTR displayed a repeated 32 bp core motif in the center of the repeat, which was flanked by similar variable motifs. A total of 14 alleles were characterized based on combinations of segments, which showed post-zygotic and inter-individual variation, with up to 6 alleles in a single subject. Somatic variation occurred in ∼24% of cases. In this hypervariable region, we found a clustering of transcription factor binding sites with strongest sequence similarity to mouse Foxg1 transcription factor binding motif. This study describes a VNTR with sequence properties of an enhancer that displays post-zygotic and inter-individual genetic variation. This element is within a locus containing four related cytokine receptors: *IFNAR2*, *IL10Rβ*, *IFNAR1* and *IFNGR2*, and we hypothesize that it might function in transcriptional regulation of several genes in this cluster. Our findings add another level of complexity to the variation among VNTR-based enhancers. Further work may unveil the normal function of this VNTR in transcriptional control and its possible involvement in diseases connected with these receptors, such as autoimmune conditions and cancer.

## Introduction

The Encyclopedia of DNA Elements (ENCODE) project has recently characterized regions of transcription, transcription factor association, and chromatin structure suggesting biochemical functions for up to 80% of the human genome [1]. Repetitive DNA sequences with shorter repeat-motifs fall into two main categories; interspersed repeats and variable number tandem repeats (VNTR). The interspersed repeats are remnants of transposons and are more abundant than VNTRs [2]. In VNTRs, repeat-motifs are positioned right next to each other. They are called microsatellites, with repeat-motifs <10 bp, and minisatellites with repeats ≥10 bp. The VNTRs are sometimes extremely variable with *de novo* mutation rates up to 10^−3^ per locus per cell division and their mutation rates therefore typically exceed mutation rates of CNVs and SNPs by factor 10 to 100,000 [2]. Although historically considered as part of junk-DNA, VNTRs can affect human phenotype. For example, disorders such as Fragile X syndrome [3], spinobulbar muscular atrophy [4], and Huntington disease [5] have all been associated with specific unstable repeat expansions within genes (reviewed in [2], [6], [7]). VNTRs outside of genes have also been found to influence gene expression and phenotype. For instance, it is established that variants of a repeat in the promoter of the serotonin transporter (*SLC6A4*) are associated with expression levels and susceptibility to anxiety-disorder [8]. Similarly, VNTR variation in the promoter of nitric oxide synthase 2 (*NOS2*) alter expression and is associated with diabetic retinopathy [9]. In these above examples, the phenotypically important VNTRs were located in promoters, but gene expression can also be affected by distantly located VNTR-based enhancers. For example, variable allele length of a 5′-VNTR of the human insulin gene (*INS*) are associated with gene expression levels both *in vitro* and *in vivo*, and predispose to, among others, insulin-dependent diabetes mellitus [10], [11]. Importantly, the same VNTR region has also been found to affect the expression of another gene (*IGF2*), located downstream relative to *INS* [12]. These results provide an important proof of concept by showing that VNTRs with embedded enhancer properties can influence expression levels of distantly located genes (reviewed in [13]).

Structural changes in the genome (such as deletions, duplications/insertions, translocations, inversions and complex rearrangements) have been identified as a major type of inter-individual variation [14]. The best studied subset of structural changes, involving variation in the copy number DNA segments, are referred to as copy number variation (CNV). The rate of *de novo* formation for CNVs has been estimated to exceed the corresponding rate for single nucleotide polymorphisms (SNPs) [15], [16]. Analyses of various types of human genetic variation performed hitherto are dominated by comparisons of different people. Little attention has been paid so far to analysis of acquired during life-time differences between somatic cells from different tissues of the same person (i.e. post-zygotic variation, or mosaicism) [17]. The definition of post-zygotic variation is the presence of genetically distinct populations of somatic cells in an individual derived from a single zygote. The paucity of studies addressing post-zygotic genetic variation is remarkable, especially in view of the fact that many common disorders are apparently not a result of inheritance of defective allele(s) from the parents [18]. Reviews suggest that post-zygotic mosaicism is understudied and consequently underestimated [17], [19], [20], [21], [22]. Moreover, predictions indicate that the somatic variation must be widespread [23], [24]. Given the high *de novo* mutation rates for chromosomal regions with CNVs and VNTRs, such loci should also be somatically variable and studies using monozygotic (MZ) twins, aging human cohorts and differentiated tissues point towards the importance of this aspect of human genetics [25], [26], [27], [28].

We describe a somatically variable VNTR with a repeated motif of 32 bp on chromosome 21q22.11. This VNTR is located in a region containing four related cytokine receptor genes, namely *IFNAR2*, *IL10Rβ*, *IFNAR1* and *IFNGR2*. We show that this locus is somatically variable using cohorts of monozygotic twins as well as multiple tissues from single subjects and that it has sequence properties suggesting it to be an enhancer.

## Methods

### Cohorts of studied subjects

Blood samples from 63 pairs of monozygotic (MZ) twin pairs were studied. Of these, 22 MZ pairs were young (age 3–43) [28]. The remaining MZ twins were older than 60 years and derived from the Swedish Adoption Twin Study of Aging (SATSA) from the population-based Swedish Twin Registry [28]. Moreover, 21 patients with breast cancer were included, where we studied blood, primary tumor and normal breast tissue from the same affected breast.

### Ethics Statement

The study is approved by the Regional Research Ethics Committee in Uppsala. The collections of human samples at all involved centers (Karolinska Institutet, Sweden; NIH, USA and Oncology Center, Bydgoszcz, Poland) have been approved by their respective Research Ethics Committee. Each studied adult subject has provided written informed consent. Written informed consent was obtained from the caretakers/guardians on the behalf of the minors/children that participated in this study.

### Illumina SNP genotyping

All samples were genotyped on Illumina 610-array or 660W SNP arrays, representing a similar collection of probes (www.illumina.com/support/array/array_kits/human610-quad_beadchip_kit.ilmn and www.illumina.com/support/array/array_kits/human660w-quad_dna_analysis_kit.ilmn). Two-hundred nanogram of genomic DNA was genotyped using Illumina-human-610 and -660W arrays according to standard protocols [29], as previously described [28]. The results were analyzed for confirmation of monozygozity for each twin pair and for all samples from the same subject. The expected values for different tissues from the same person should be as genotypic concordance for monozygotic twins (>99.9% identical). We analyzed Illumina files by Nexus-Copy-Number program (BioDiscovery, CA, USA).

### Bioinformatic analysis of the locus between *IL10Rβ* and *IFNAR1* genes

The locus showing variation was analyzed for repeated elements with the RepeatMasker [30] and tools such as MREPS [31] and Tandem Repeats Finder [32]. Apart from describing the tandem repeat sequence, these analyses also defined non-repetitive regions in this locus that could be used for primer design and definition of alignment-anchors.

### Polymerase chain reaction using genomic DNA

All PCRs were performed in 25 µl reactions comprising 10 ng gDNA, 0.4 mM each dNTP (Saveen Werner, Sweden), 2 mM MgCl_2_ (Invitrogen, Carlsbad, USA), 0.4 µM of primers 1 and 8 (primer 1, 5′-CCTAACAGCTGGATAGATTGCC-3′ and primer 8, 5′-CCATGCGTGTATATTCCATACG-3′), 1× PCR buffer (Invitrogen) and 0.04 U/µl Platinum Taq DNA polymerase (Invitrogen). Phusion high-fidelity DNA polymerase (New England BioLabs; cat. No M0530L) was also used according to the manufacturer's recommendations. The PCR products were analyzed on 1% agarose gel.

### TA cloning, plasmid purification

For sub-cloning in pCR2.1-TOPO vector (Invitrogen), 2 µl of PCR product was used. Up to 50 clones per single PCR product were picked and cultured in LB medium containing 50 µg/ml kanamycin. The plasmids were purified using QIAprep miniprep (Qiagen, Hilden, Germany). To confirm the presence of insert, 500 ng of plasmids were digested with EcoRI enzyme (NEB, Ipswich, USA) and analyzed on 1% agarose gel.

### Sanger sequencing

225 ng of plasmid DNA and 1.6 µM sequencing primer were used in 10 µl reactions with 4 µl BigDye® Terminator v3.1 (Applied Biosystems, Foster city, USA). Each sequencing reaction was done in triplicate on ABI 3730×l machine. Sequencing primers were: primer 2, 5′-GAATCGCTTGAACCCGGAAGG-3′; primer 3, 5′-CAGGAGAATCGCTTGAAC-3′; primer 4, 5′-CCTGGGTAACACAGCGGAAATCC-3′; primer 5, 5′-GTTGTGGTGAGCCGAGATCG-3′; primer 6, 5′-ATACGTATATATTCCATACT-3′; primer 7, 5′-TATATTCCATACGTATATATT-3′; M13 forward, 5′-GTAAAACGACGGCCAG-3′; and M13 reverse, 5′-CAGGAAACAGCTATGAC-3′. Sequences were aligned and manually inspected using the CodonCode-Aligner software (http://www.codoncode.com/aligner/index.htm). We implemented the Phrap-algorithm for accuracy of the assembly, as recommended for repeat-rich sequences. For a sequence to enter further analysis, at least one sequence-read had to go through both of the alignment-anchors (i.e. non-repeated flanking sequences), to avoid erroneous alignments. After alignment the ‘compare contigs’ option in the CodonCode-Aligner was used to compare the consensus sequences of the identified alleles. The sequenced alleles are deposited in the GenBank (accession numbers JQ904024 through JQ904030).

### Analysis of tentative enhancer element and CpG islands

We used the Enhancer Element Locator (EEL) computer program [33] to identify suspected enhancer elements in the locus between the *IL10Rβ* and *IFNAR1* genes. Conserved non-coding elements (CNEs) were predicted by EEL-program using the JASPAR CNE matrix profiles [34], [35]. This is a collection of 233 matrix profiles from human conserved non-coding elements for analysis of transcription factor binding sites (TFBS) and enhancer elements. TOMTOM Motif Comparison Tool was also used in analysis of the core sequence of the hypervariable region for similarities with known motifs for transcription factor binding (http://meme.nbcr.net/meme/cgi-bin/tomtom.cgi) [36]. To analyze the GC-content and predict CpG islands in the region we used the CPGPLOT-software (http://www.ebi.ac.uk/Tools/emboss/cpgplot/).

## Results

### Illumina SNP genotyping suggests a post-zygotic and inter-individual variation in the locus between *IL10Rβ* and *IFNAR1*

**Figure 1:**
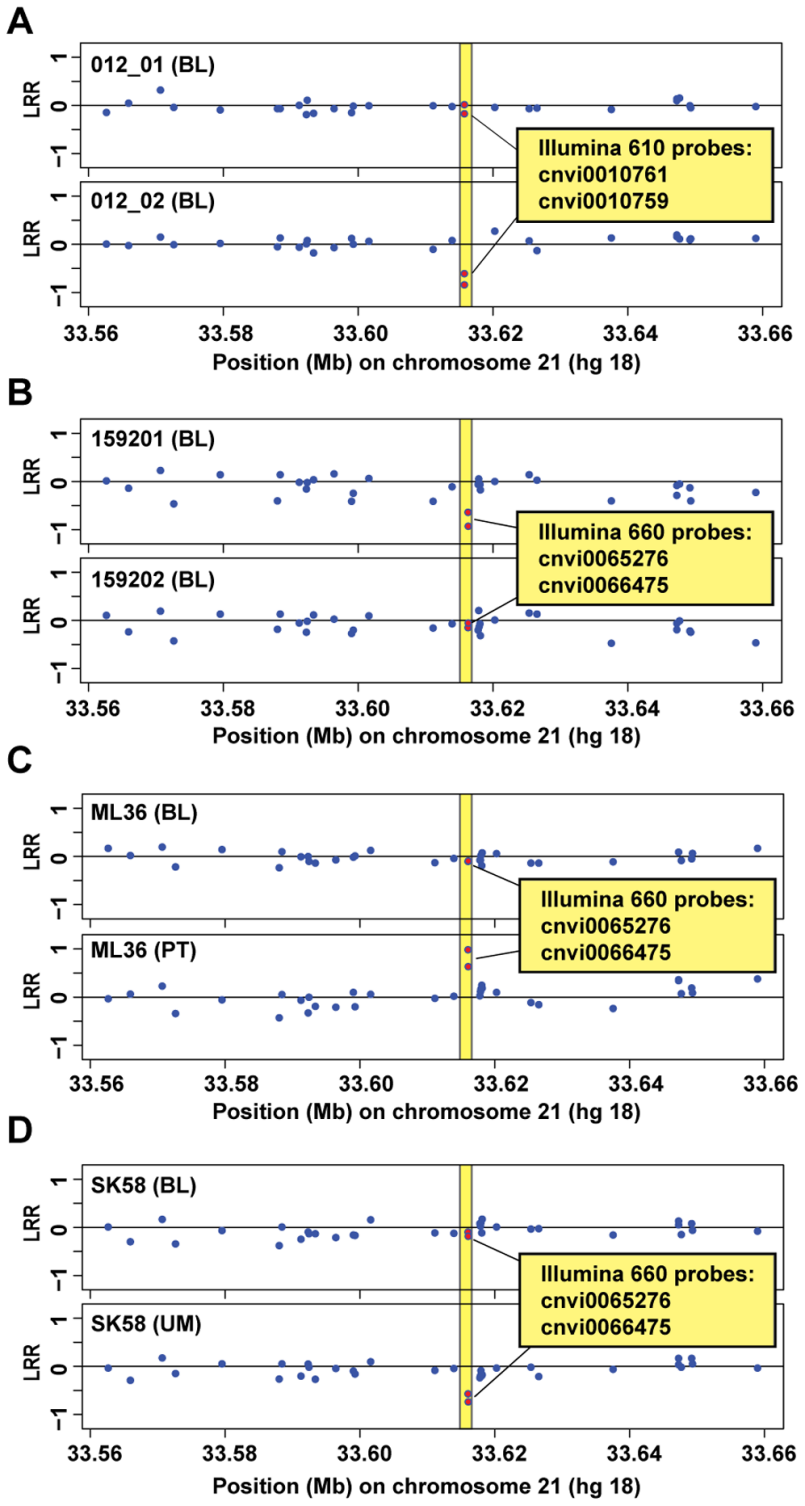
Indications of post-zygotic copy number variation in a region between *IL10Rβ* and *IFNAR1*. Results from eight Illumina genotyping experiments are shown using blood DNA from two pairs of monozygotic twins (panel A for twin 012_01 versus co-twin 012_02, and in panel B for twin 159201 versus co-twin 159202) and two unrelated individuals, where two different tissues were analyzed from each subject (Panels C and D; subjects ML36 and SK58, respectively). Abbreviations BL, PT and UM indicate peripheral blood DNA, primary breast tumor and healthy morphologically normal breast tissue from a patient affected with breast cancer, respectively. Illumina 610 or 660W SNP arrays were used, which also contain so called “intensity only probes” (often with cvni-prefix), only useful for copy number analyses. Therefore, only Log R Ratio (LRR) windows of Illumina experiments are shown here, since the B Allele Frequency (BAF) values are not informative for this type of probes. LRR values below and above zero suggest a deletion or a gain, respectively. The four array probes showing variation between the studied samples are labeled as red dots in yellow fields.

We initially genotyped blood DNA from 63 monozygotic (MZ) twin pairs using Illumina 610 or 660W SNP arrays. Twenty-two MZ pairs were genotyped using 610 array and 41 MZ pairs were genotyped using 660W SNP arrays and each MZ pair was treated as a single case for analysis of post-zygotic variation. Because nuclear genomes of MZ twins are identical at conception, they represent a good model for studying post-zygotic variation. The results were analyzed using Nexus-Copy-Number-Pro-software and the region between *IL10Rβ* and *IFNAR1* genes in 21q22.11 attracted our attention as it showed a frequent variation. Twenty eight out of 63 MZ twin pairs (44.4%) showed clear-cut differences in fluorescent intensity signal as measured by Log R ratio (LRR), either for two consecutive probes on 610-array (cnvi0010759 and cnvi0010761, at positions of 33615750 bp and 33615748 bp, respectively) or for two other adjacent probes on 660W-array (cnvi0065276 and cnvi0066475, at positions 33616087 bp and 33616031 bp, respectively) (Fig. 1). For instance, the two probes on 610 array indicated deletion in this region in blood DNA of twin 012_02, when compared to its co-twin and the two probes on 660W SNP array showed deletion in blood DNA of twin 159201, compared to its co-twin (Fig. 1A and B).

We further expanded our study by 660W-array SNP genotyping of multiple tissues from 21 patients diagnosed with breast cancer, where peripheral blood, primary breast tumor, and healthy morphologically normal breast tissue from the breast affected with primary tumor were analyzed. Here we counted each patient as one case for analysis of post-zygotic variation. Clear differences in LRR values were observed also in this cohort for two probes (cnvi0066475 and cnvi0065276) when comparing different tissues of the same patient in 13 out of 21 cases (61.9%) (Fig. 1C and D). Thus, in summary for both cohorts of MZ twins and breast cancer patients, Illumina SNP beadchips suggested a post-zygotic mosaicism in 41 out of 84 (48.8%) cases, in the locus between *IL10Rβ* and *IFNAR1* genes represented by the four consecutive array probes. Statistical analysis on this relatively limited sample size failed to reject the hypothesis of no association between cancer diagnosis and presence of post-zygotic mosaicism (not shown).

### Post-zygotic and inter-individual mosaicism of a presumptive enhancer embedded in a variable number of tandem repeats (VNTR)

**Figure 2:**
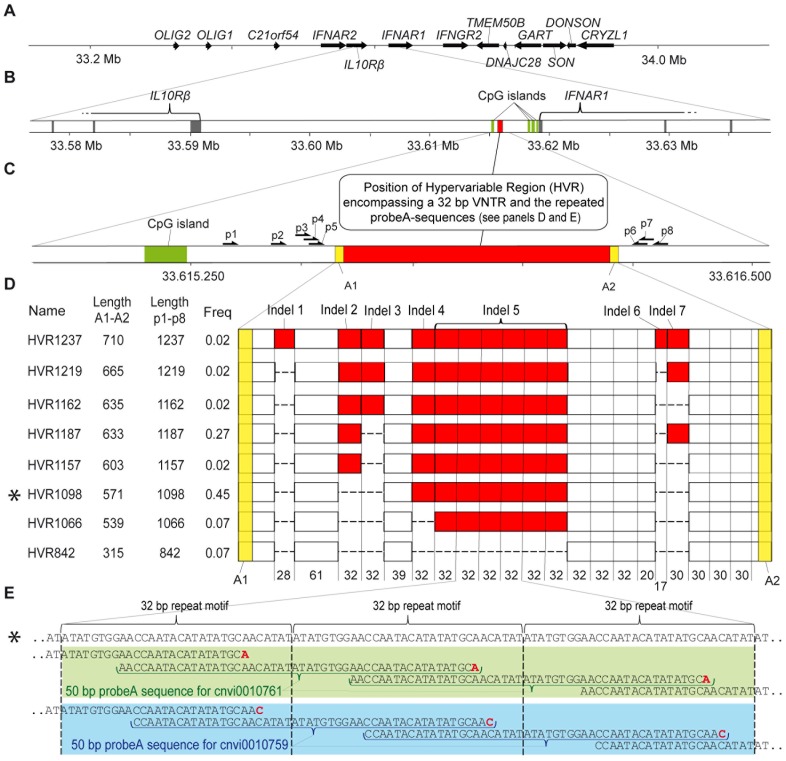
Graphical summary of variation in a presumptive regulatory VNTR containing region. Panel **A** shows an overview of approximately 2 Mb locus on 21q, around four genes encoding functionally related receptors; *IFNAR2*, *IL10Rβ*, *IFNAR1* and *IFNGR2*. Panel **B** is zooming on the position of the hypervariable region (HVR, red box), which is located approximately 4 kb upstream from the transcription start site of the *IFNAR1* gene and is flanked by CpG-islands (green boxes). The last three and the first three exons of *IL10Rβ*, and *IFNAR1*, respectively, are shown as grey boxes. Panel **C** is showing the size and position of HVR according to the most common allele (HVR1098, see below panel D) in relation to the CpG island. Positions of PCR and sequencing primers used in the analysis of the locus are also displayed. Yellow boxes indicate the position of the non-repetitive anchor 1 (A1) and anchor 2 (A2) sequences, that are immediately flanking the repeated segments and were used for alignments of sequence reads. Panel **D** shows a summary of eight HVR-alleles from the studied samples, which were identified based on Sanger sequencing results of PCR fragments sub-cloned in plasmids. The displayed alleles are ordered from longest to shortest according to size from anchor 1 (A1) to anchor 2 (A2) sequences. Summary of sizes for all 14 different HVR-alleles is shown in Table 1. Sizes of fragments (in base pairs) are given between non-repetitive A1 and A2 sequences and between primers p1 and p8, which were used for PCR amplification from genomic DNA. Asterisk (*) indicates the most frequent allele (HVR1098), which is in agreement with the reference sequence according to NCBI sequence build 36.3. The allele frequency shown here is taking into account only the nine alleles, where the entire sequence could be unequivocally determined using Sanger sequencing. The most common variation encompasses the variable number of 32 base pair segments; i.e. indel 2, indel 3, indel 4, and indel 5. The latter indel 5 is composed of 6 repeated 32 base pair segments (HVR1066). However, there are also indels containing shorther segments; e.g. indel 1, indel 6 and indel 7. Panel **E** illustrates the positions of two of the four probes from Illumina beadchips, which are aligned onto the NCBI reference sequence for this locus (top sequence with an asterisk, representing HVR1098). The two probes shown here are from Illumina 610 SNP array; cnvi0010761 (green) and cnvi0010759 (blue). All four Illumina probes from Figure 1, which were used for initial identification of variation in this region are located within hypervariable region. As shown here for two of these four probes, the probeA sequences (as called by Illumina and used for capturing of genomic DNA on beadchips) are shifted only by two bases. The core 32 bp repeat motif is shown in brackets.

Application of repeat finding programs on the reference sequence encompassing locus between the *IL10Rβ* and *IFNAR1* genes, revealed that the four above mentioned Illumina probes are located within a previously not characterized variable number of tandem repeats (VNTR) (Fig. 2). The NCBI reference sequence (build 36.3) displayed a 32 bp core motif repeated seven times in the center of the tandem repeat locus and this core was flanked by several similar sequence motifs (Fig. 2D, see HVR1098). This VNTR segment was further embedded in a region very rich in other types of common repeats (not shown), which made it challenging to design PCR- and sequencing-primers in the region around the VNTR segment. The primers that generated reliable results are shown in Figure 2C. The only primers that reproducibly generated a PCR product from genomic DNA were primers 1 and 8.

**Figure 3:**
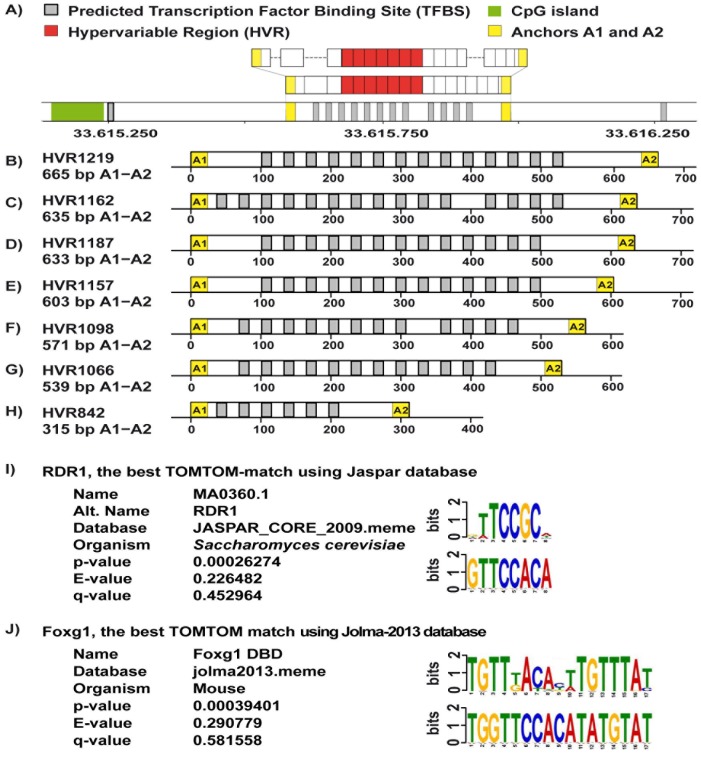
Analysis of the transcription factor binding sites (TFBSs) for the sequenced HVR-alleles. Panel **A** shows graphical representation of the most frequently occurring allele in our data set (HVR1098) with identical distribution of in-dels as compared to the NCBI reference genome build 36.3 (see also Fig. 2B–D). The HVR region is flanked by the non-repetitive sequences referred to as anchor A1 and A2 (yellow boxes) that were used for alignment of sequence reads. Green box illustrates the position of a CpG island and the overall layout of this figure is analogous to Fig. 2. Analysis using the EEL-software (Enhancer Element Locator [33]) of ∼1.2 kb of the NCBI reference sequence upstream from the transcription start site of the *IFNAR1* gene on chromosome 21, showing a clustering of TFBSs in the core of the HVR. Grey boxes indicate the positions of the predicted TFBSs in the region and they were determined by the CN0062.1 matrix profile (from ref. [35]) with the consensus sequence 5′- AATTGCTTCCAGATG. Panels **B–H** show the predicted TFBSs in sequenced alleles HVR1219, HVR1187, HVR1162, HVR1157, HVR1098, HVR1066 and HVR842, identified using the matrix profile CN0062.1. Panel **I** shows results of the best match for Saccharomyces *cerevisiae* transcription factor RDR1 (for **R**epressor of **D**rug **R**esistance **1**) with TOMTOM Motif Comparison Tool [36]. Panel **J** illustrates TOMTOM-based analyses of mouse and human database of transcription factor binding sites [39], which revealed the best match to the mouse transcription factor Foxg1 binding motif.

**Figure 4:**
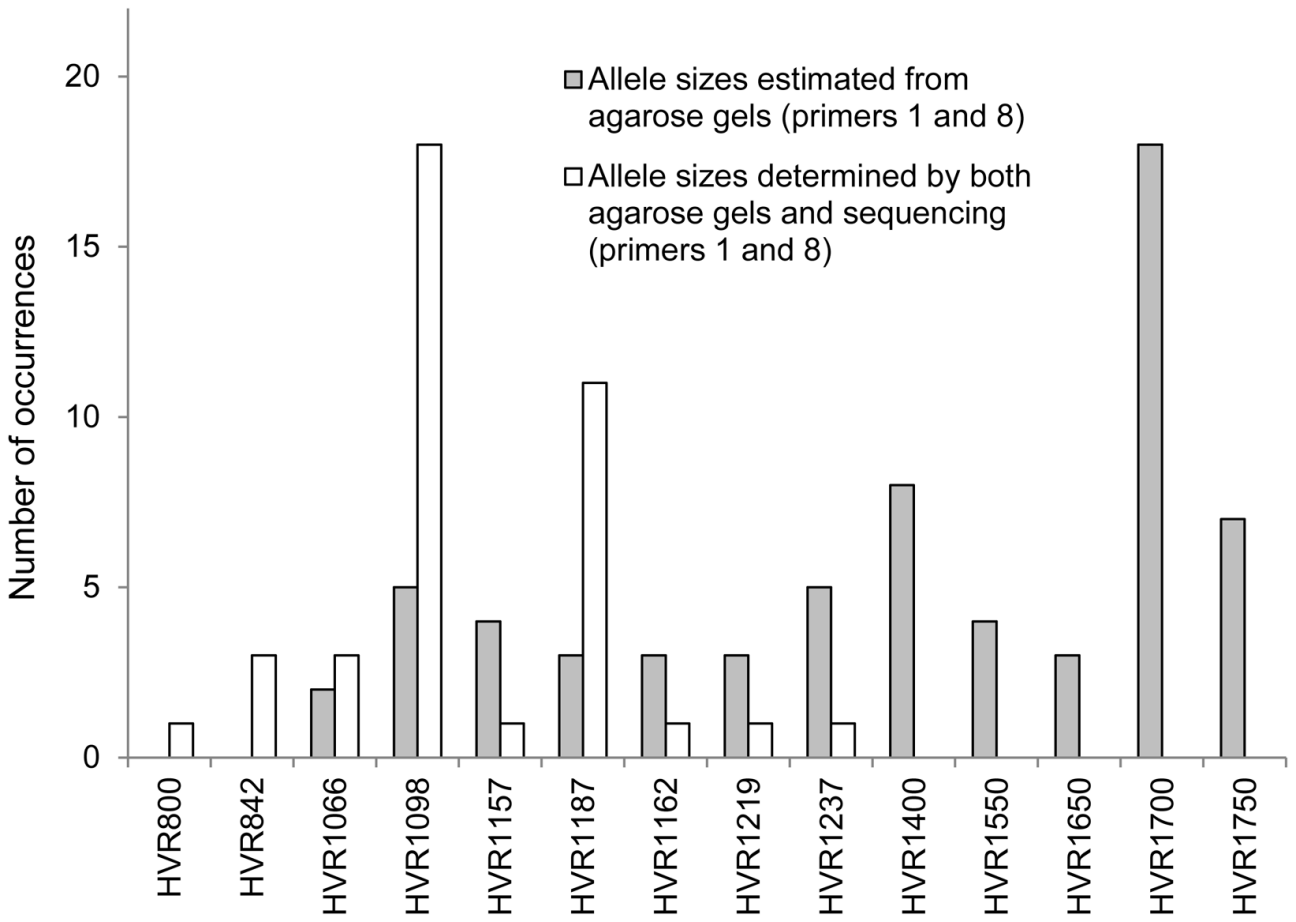
The frequency distribution of the 14 alleles identified for the hypervariable region. The white bars represent alleles defined by both agarose gels and Sanger sequencing. The grey bars denote alleles that were characterized by estimation of their sizes from agarose gels. All alleles were defined based on the analysis between primers 1 and 8 (see Fig. 2 and Table 1).

**Figure 5:**
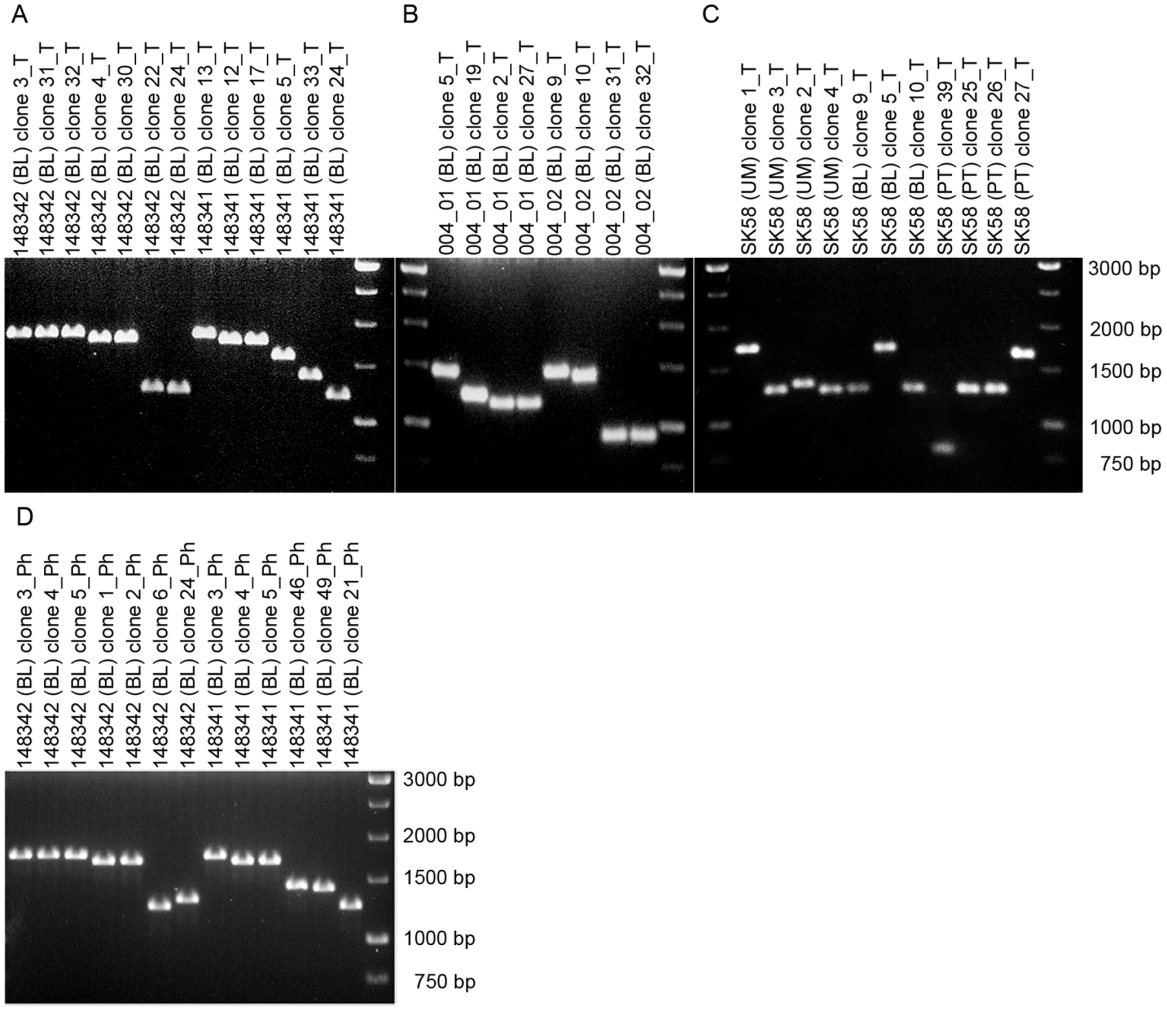
Variable length of alleles within hypervariable region showing post-zygotic variation. Panel **A** shows post-zygotic mosaicism in healthy and phenotypically concordant monozygotic twin pair 148341/148342, with five alleles observed in twin 148341, and three alleles present in co-twin 148342. Similarly, panel **B** displays post-zygotic variation in another monozygotic twin pair 004_01/004_02. In total 5 different alleles are shown on this gel and only one of them is overlapping between both twins. Panel **C** illustrates post-zygotic mosaicism in breast cancer patient SK58. There are three different alleles in DNA from morphologically normal breast tissue (UM), two alleles in blood cells (BL) and three alleles in primary tumor (PT). In panels **A**, **B** and **C**, Taq DNA polymerase was used for initial PCR amplification from genomic DNA, as indicated by suffix “T” in the ID of each plasmid clone. In panel **D**, Phusion DNA polymerase confirmed post-zygotic mosaicism in monozygotic twin pair 148341/148342, as indicated by suffix “Ph” in the ID of each plasmid clone. The length of inserts in all plasmid clones was estimated after EcoRI digestion releasing the insert, and using 1% agarose gel. BL, PT and UM indicate peripheral blood DNA, primary breast tumor and healthy morphologically normal breast tissue from a patient affected with breast cancer, respectively.

For the PCR-based validation of array results we selected a representative series of 17 cases where Illumina results suggested post-zygotic variation (Table 1, cases 1–17, Fig. 3, 4, 5). These encompass MZ twin pairs (9 cases) and samples from tissues of breast cancer patients (8 cases). The list of subjects taken for validation included also 7 additional samples from unrelated control subjects, where only one tissue was studied. We approached validation in this region by PCR amplification from genomic DNA with primers 1 and 8. This was followed by sub-cloning of the products into plasmids, preparation of plasmid DNA from 20–50 bacterial clones for each PCR reaction, analysis of inserts and Sanger sequencing using the plasmid DNA as template. It was apparent from the initial PCR reactions with primers 1 and 8 that there was a considerable variation in the length of amplified fragments. The sequencing was performed using primers 2 through 7 (Fig. 2) as well as with plasmid-based primers, using all subcloned fragments that were of different size, compared to the most common allele (see below). The sequence assembly was performed separately for each subcloned fragment and was guided by the use of anchor 1 (A1) and anchor 2 (A2) sequences (Fig. 2C and D), which were non-repetitive and located close to the cassette of tandem repeat motifs. The Sanger sequencing confirmed the variation and established nine fully sequenced alleles, with at least two high-quality forward and reverse sequence reads, showing sufficient overlap to assure the correct assembly across multiple similar sequence motifs. These sequences are deposited in the GenBank database (accession numbers JQ904024 through JQ904030). We named each of the alleles as HVR (for **h**yper**v**ariable **r**egion), followed by the number of nucleotides in the sequence between primers 1 and 8. Among the fully sequenced alleles, the most abundant one (HVR1098, allele frequency ∼45%, Fig. 2D) was in agreement with the genome reference sequence. In addition to HVR1098, we established the full sequence for five longer alleles (HVR1157, HVR1162, HVR1187, HVR1219 and HVR1237) and three shorter (HVR1066, HVR842 and HVR800) (Fig. 2 and Table 1). However, the limits of current sequencing technology did not permit establishing of the full-length sequence for the five additional longer HVR alleles (based on the same stringent quality control of sequence reads that are aligned between anchors 1 and 2 segments) and their sizes were therefore estimated from agarose gels (Fig. 5). We also considered a possibility that the observed variation might be due to PCR artifacts. We addressed this by using different DNA polymerase with proof-reading activity. Selected samples (Fig. 5) were a subject to PCR amplification using (in addition to Taq polymerase) Phusion high-fidelity DNA polymerase starting from genomic DNA and the results showed the same pattern of post-zygotic variation.

**Table 1:** Size and distribution of 14 HVR-alleles identified by sequencing and gel electrophoresis.

Case no.	Subject ID	Tissue	HVR800	HVR842	HVR1066	HVR1098	HVR1157	HVR1187	HVR1162	HVR1219	HVR1237	HVR1400	HVR1550	HVR1650	HVR1700	HVR1750	Mosaic cases (no. of different alleles)
1	PF27*	BL			•/×												
	PF27*	UM			•/×												
	PF27*	PT			•/×												
2	SK58*	BL						•/×							•		2
	SK58*	UM						•/×			•/×				•		3
	SK58*	PT	•/×					•/×							•	•	4
3	JM43*	BL				•/×											
	JM43*	UM				•/×											
	JM43*	PT				•/×											
4	KJ42*	BL						•/×							•		
	KJ42*	UM						•/×							•		
5	PK6*	BL						•/×							•		
	PK6*	UM						•/×							•		
	PK6*	PT						•/×							•		
6	NM48*	BL				•/×						•					
	NM48*	UM				•/×						•					
	NM48*	PT				•/×						•					
7	KU25*	BL				•											
	KU25*	UM				•											
	KU25*	PT				•/×											
8	ML36*	BL				•	•	•/×						•	•	•	6
	ML36*	UM					•	•/×								•	3
9	159201**	BL		•/×		•/×											2
	159202**	BL				•/×											1
10	004-01**	BL		•/×		•/×			•/×		•	•	•				6
	004-02**	BL		•/×								•	•				3
11	030-01**	BL				•/×											
	030-02**	BL				•/×											
12	148341**	BL				•/×					•		•		•	•	5
	148342**	BL				•/×									•	•	3
13	2123411**	BL					•		•	•/×							3
	2123412**	BL					•		•	•	•						4
14	21801**	BL			•	•/×			•	•	•						5
	21802**	BL				•/×					•						2
15	183411A**	BL						•							•	•	3
	183412A**	BL													•		1
16	188831**	BL						•		•					•	•	4
	188832**	BL						•							•		2
17	169101A**	BL				•						•					2
	169102A**	BL										•					1
18	F1	BL			•	•/×											
19	M1	BL										•			•		
20	Stan	BL						•/×					•				
21	01DM	BL												•	•		
22	22LG	BL												•	•		
23	25AK	BL				•											
24	PE129	BL				•/×	•/×										

The •/× indicate that the size of the allele was determined by both agarose gel images and sequencing, whereas filled circles (•) denote that the allele size was estimated from agarose gel images.

The two most common alleles (HVR1098 and HVR1700) are highlighted in bold and underlined text.

The sample indicated by a single asterisk (*) are from breast cancer patients. BL, PT and UM indicate peripheral blood DNA, primary breast tumor and healthy morphologically normal breast tissue from a patient affected with breast cancer, respectively.

The samples indicated by two asterisks (**) are monozygotic twin pairs.

It is noteworthy that we validated a relatively high percentage (10 out of 17; 58.8%) of cases that were suspected to contain post-zygotic genetic variation in the studied locus from the Illumina data. When extrapolated to all subjects included in our study, somatic variation in this VNTR locus is approximately 24%, which is a high number for a locus displaying post-zygotic variation, when compared with the literature [17]. We further calculated the total number of times, for all samples and all cases combined, that each of the 14 HVR-alleles occurred in our study (Fig. 4). The most common allele was HVR1098 that was fully sequenced and was in agreement with NCBI reference sequence. The second most common was HVR1700 and its full sequence still remains to be determined. Table 2 shows the summary of all cases, where Illumina data could be validated. To explain as to why the validation of Illumina results was unsuccessful for approximately 40% of cases, we analyzed the “probeA”-sequences that are used for synthesis of probes attached to beads and capture of genomic DNA on Illumina beadchips. Global genome *BlastN* searches for similarities of “probeA”-sequences for cnvi0010759, cnvi0010761, cnvi0065276 and cnvi0066475 showed that these are not unique (not shown), as they match to multiple regions in the human reference sequence and this is likely the reason for the not fully predictable behavior during genotyping. We presume that the results of Illumina copy number genotyping for these “probeA”-sequences is the sum of variation at the HVR region and possible variation that occurs in other regions of the genome with similarities to the “probeA”-sequences. This implies that the sole analysis of Illumina beadchip data from these four probes is not a sufficiently reliable tool for typing of the copy number status within the HVR in the locus between *IL10Rβ* and *IFNAR1*. Nevertheless, it was a good initial tool for variation detection.

**Table 2:** Summary of validation of somatic variation in the *IFNAR1* locus.

Subject ID	Analysed tissues	Mosaicism indicated by Illumina platform?	Illumina results verified?	Validation method
SK58*	BL, UM, PT	Yes	Yes	Gel+Seq
ML36*	BL, UM, PT	Yes	Yes	Gel+Seq
KJ42*	BL, UM	Yes	No	Gel+Seq
NM48*	BL, UM, PT	Yes	No	Gel+Seq
KU25*	BL, UM, PT	Yes	No	Gel+Seq
PF27*	BL, UM, PT	Yes	No	Gel+Seq
JM43*	BL, UM, PT	Yes	No	Gel+Seq
PK6*	BL, UM, PT	Yes	No	Gel+Seq
004_01/004_02**	BL	Yes	Yes	Gel+Seq
159201/159202**	BL	Yes	Yes	Gel+Seq
21801/21802**	BL	Yes	Yes	Gel+Seq
183411A/183412A**	BL	Yes	Yes	Gel
148341/148342**	BL	Yes	Yes	Gel+Seq
169101A/169102A**	BL	Yes	Yes	Gel
188831/188832**	BL	Yes	Yes	Gel
2123411/2123412**	BL	Yes	Yes	Gel+Seq
030_01/030_02**	BL	Yes	No	Gel+Seq

Summary of Illumina SNP genotyping, which suggested structural variation within the hypevariable region and results from subsequent confirmation using Sanger sequencing and agarose gel electrophoresis. One (*) and two (**) asterisks after the subject ID indicate patients with breast cancer and pairs of monozygotic twins, respectively. BL, UM and PT stand for DNA from peripheral blood cells, healthy morphologically normal breast tissue from a patient affected with breast cancer and primary breast tumor, respectively. “Seq” indicate that the somatic mosaicism was verified by Sanger sequencing while “Gel” shows that it was confirmed by estimation of allele sizes from agarose gel.

The sequence of the segment between *IL10Rβ* and *IFNAR1* was also analyzed using multiple bioinformatic tools for the content of functional elements important for gene transcription. We identified features suggesting that the HVR might influence gene transcription from this locus. The ELL-program found a clustering of suspected transcription factor binding sites approximately 4 kb upstream from *IFNAR1* transcription start site, suggesting an enhancer. This presumptive enhancer element was overlapping with the hypervariable region (Fig. 3) and different alleles had different number of clusters with binding properties for transcription factors. Specifically, EEL program [33] identified similarity between HVR sequences and CN0062.1 matrix profile [35] with the consensus sequence 5′-AATTGCTTCCAGATG. This 15 bp sequence motif had 4 mismatches to the core of tandem repeat in the HVR sequences identified among the nine sequenced alleles. Using TOMTOM Motif Comparison Tool [36] we further analyzed 15 bp of our core HVR sequence (5′-TATTGGTTCCACATA), with or without additions of 5, 7 and 10 bases on both sides flanking the above sequence. From analyses of Jaspar database, four transcription factors of Saccharomyces *cerevisiae* (RDR1, HAP2, OPI1 and HSF1) consistently showed the strongest scores. Results of the best match for the transcription factor RDR1 (for **R**epressor of **D**rug **R**esistance **1**) [37], [38] are shown in Fig. 3I. The RDR1 zinc cluster protein is a member of Gal4p family of transcriptional regulators and its normal function in yeast is the transcriptional control of multidrug resistance. Similar TOMTOM-based analyses of recently published mouse and human database of transcription factor binding sites [39] revealed strongest match to the mouse transcription factor Foxg1 binding motif (Fig. 3J). Forkhead-box (FOX) family of transcription factors represent a large group of proteins with crucial roles in development and metabolism, which were studied in many species. The human genome encodes 43 FOX proteins [40], [41], [42]. The *FOXG1* is crucial for normal brain development [43] and loss-of-function mutations in *FOXG1* cause an atypical form of Rett syndrome [44]. On the other hand, overexpression of *FOXG1* is implicated in cancer development [45], [46]. In summary, the motif analyses suggest that the HVR locus might function as an enhancer but we have not unambiguously identified its protein binding partners. Further studied are needed to address this question.

Furthermore, mining of the ENCODE dataset [1] (http://genome.ucsc.edu/ENCODE/) supported a role for the 1.45 kb region (displayed in Fig. 2C) as a suspected enhancer element (Fig. S1). The ChIP-seq ENCODE data was used, in combination with the ChromHMM and Segway programs to perform the segmentations in six cell lines. Results from three cell lines (GM12878, HeLa-S3 and HepG2) were consistent with enhancer predictions. In agreement with the above, ENCODE data tracks representing the set of open chromatin elements and signals based on DNase-seq results suggest multiple DNaseI hypersensitivity clusters in CD20+ cells and four additional cell lines (Fig. S1). Analyses also showed the presence of a CpG island just upstream the predicted enhancer element (Figs. 2 and 3). Thus, in summary, the above observations suggest a functional gene-regulatory role of the studied locus and that the VNTR we describe should be studied further for functional consequences on gene transcription.

## Discussion

Post-zygotic variation has been estimated to ∼1% in young subjects routinely analyzed in the course of genetic counseling [47]. Our recent analyzes in population-based aging cohort showed that ∼3.5% of people older than 60 years carry mega-base range post-zygotic rearrangements [28]. Our current results suggest that the VNTR locus studied here has a considerably higher rate of post-zygotic variation, as ∼24% of cases in this study have been estimated to display post-zygotic differences. Furthermore, this number is likely an underestimate, as a limited number of specimens from different tissues were studied for each case. These results are in agreement with the estimates suggesting that *de novo* mutation rate for VNTRs is higher than the corresponding rates for other types of structural rearrangements [2]. The inter-individual copy number variation in the VNTR locus studied here has been noticed previously, but was not explored in detail [14] (supplemental data; locus ID CNVR7995). Conrad et al. 2010 applied two variation detection methods; first was Nimblegen genotyping on 42M genome-wide tiling-path array. Results from this array were validated by 105K Agilent array designed to target the variable loci only. The CNVR7995 in Conrad et al. is one among 8599 inter-individually variable loci. Although CNVR7995 was shown variable among different people, it could not be unambiguously genotyped by further array-CGH experiments using 450 subjects; i.e. placed in a one class of genotypes such as heterozygous deletion, diploid state, gain of one (or more) copies and was therefore not included in the final list of 5238 validated and genotyped loci. A possible reason behind the failure in this last step could be the post-zygotic variation in CNVR7995; i.e. DNA samples studied contained a mixture of genotypes and were not possible to score unambiguously. The intriguing question emerging from the above reasoning is: Are the remaining loci (39%, 3360 loci that could not be unambiguously genotyped on the final validation platform in Conrad et al. 2010) also showing post-zygotic variation?

One of the first described and well-studied VNTR-based enhancers in the human genome is located upstream of the human insulin gene (*INS*) on chromosome 11p15. This VNTR has been shown to regulate not only the expression of the closest gene (*INS*), but also the *IGF2* gene, located downstream on the same DNA strand from *INS*, thus demonstrating the long range effect of the VNTR on gene transcription [12], [48]. The field of DNA enhancers provides additional examples regarding a capacity of the enhancers to exert their regulatory effects on genes located at large genomic distances. Enhancers can activate gene expression independent of their orientation and are commonly scattered across noncoding intervals, in extreme cases functioning at a distance of >1 Mb from their gene promoter target [13], [49]. The VNTR upstream of the *INS* gene has also attracted a considerable interest in medical genetics, as its length has been associated with type 1 and type 2 diabetes, latent autoimmune diabetes in adults (LADA), polycystic ovary syndrome as well as with size at birth [50], [51], [52], [53], [54].

The region around the presumptive enhancer studied here (Fig. 2) contains four functionally related receptor genes: interferon alpha, beta and omega receptor 2 (*IFNAR2*); interleukin 10 receptor beta (*IL10Rβ*); interferon alpha, beta and omega receptor 1 (*IFNAR1*); and interferon gamma receptor 2 (*IFNGR2*). Considering the above discussed regulatory functions for VNTR upstream of the *INS* gene and other well studied enhancers, it is reasonable to hypothesize that this tentative VNTR-based enhancer located between *IL10Rβ* and *IFNAR1* genes might be involved in regulation of transcription of not only *IFNAR1* gene, but also additional genes located in its vicinity. This issue is an important subject for future studies. There exists an extensive literature describing physiological functions for these interferon/cytokine receptor pathways and their involvement in disease-related processes [55], [56], [57], [58], [59]. For instance, type-I interferon (IFN-alpha)-induced signaling was reduced in T cells and B cells from 3 major groups of cancer patients (breast cancer, melanoma, and gastrointestinal cancer) compared to healthy controls. Type-II interferon (IFN-gamma)-induced signaling was also reduced in B cells from these cancer patient groups. These findings suggest that defects in lymphocyte IFN signaling arise in patients with common cancers, and these defects may represent a common cancer-associated mechanism of immune dysfunction [56]. Interferon alpha is also implicated in the autoimmune disease such as systemic lupus erythematosus, where there is a continuous overproduction of IFN-alpha and increased expression of IFN-alpha-regulated genes [60].

## Conclusions

This study shows post-zygotic genetic variation in a VNTR-locus, which has sequence properties of an enhancer. This variation may affect the expression of gene(s) that are under the control of this regulatory element and understanding the phenotypic consequences of both post-zygotic and inter-individual variation within this enhancer on the level of single cell and whole organism is a task for future work. Our findings of post-zygotic genetic variation add another level of complexity to the genetic variation among VNTR loci in general and in particular to the possible phenotypic consequences of variation in the VNTR-locus between *IL10Rβ* and *IFNAR1* genes. This VNTR-based presumptive enhancer is within a locus containing four functionally related cytokine receptor genes; *IFNAR2*, *IL10Rβ*, *IFNAR1* and *IFNGR2*. We hypothesize that this element might function in transcriptional regulation of not only the closest *IFNAR1* gene, but also other flanking genes. The extension of our work may unveil the normal function of this VNTR-locus in transcriptional control of genes. Furthermore, our work opens up for studies of associations of length of VNTR alleles in cohorts of patients with various immune system dysfunctions, for instance autoimmune disorders or cancer. Interferons, acting via multiple receptor genes encoded from the locus studied here, are already used in clinical treatment of patients with various conditions. Our study may also be a starting point for analysis aiming at understanding of why there often is a variable response to treatment with interferons.

## Supporting Information

Figure S1 **The results from mining of the Encode project dataset (** http://genome.ucsc.edu/ENCODE/ **).** Panel **A** shows ∼13 kb segment centered on HVR with co-ordinates chr21:34687376-34700434 (build 37/hg19) and chr21:33609246-33622304, acc. to build 36/hg18. On the right hand side, this view also includes the promoter/cis-regulatory elements and exon 1 of the *IFNAR1* gene. The three colored solid horizontal bars show results (in compressed view) from the ChIP-seq experiments in six cell lines. These were analyzed by a combination of the ChromHMM and Segway programs to perform the segmentations. Three cell lines (GM12878, HeLa-S3 and HepG2) showed “predicted weak enhancer or open chromatin cis regulatory element” in yellow. Other color codes indicate: bright red, predicted promoter region including transcription start site; light red, predicted promoter flanking region; dark green, predicted transcribed region; gray, predicted repressed or low activity region; light green, low activity region. Results from ENCODE data tracks representing the open chromatin signals based on DNase-seq experiments are shown below. Light blue bars indicate DNaseI hypersensitivity clusters in CD20+ cells, GM18507 cells, HA-sp cells, HCFaa cells and HCM cells. Panel **B** illustrates ENCODE data in greater detail for a segment of 1.45 kb, which corresponds to the region displayed in Fig. 2C (chr21:34693180-34694630, build 37/hg19; and chr21:33615050-33616500, build 36/hg18). The results from ChIP-seq experiments and data tracks representing the open chromatin signals based on DNase-seq experiments are shown in expanded view. The color codes for different predictions are the same as described above for panel A.
(TIF)
