# Supplementary figures and images for: Post-Zygotic and Inter-Individual Structural Genetic Variation in a Presumptive Enhancer Element of the Locus between the IL10Rβ and IFNAR1 Genes
Source: PLoS One. 2013 Sep 4;8(9):e67752. doi: 10.1371/journal.pone.0067752 (PMC3762855; doi:10.1371/journal.pone.0067752)

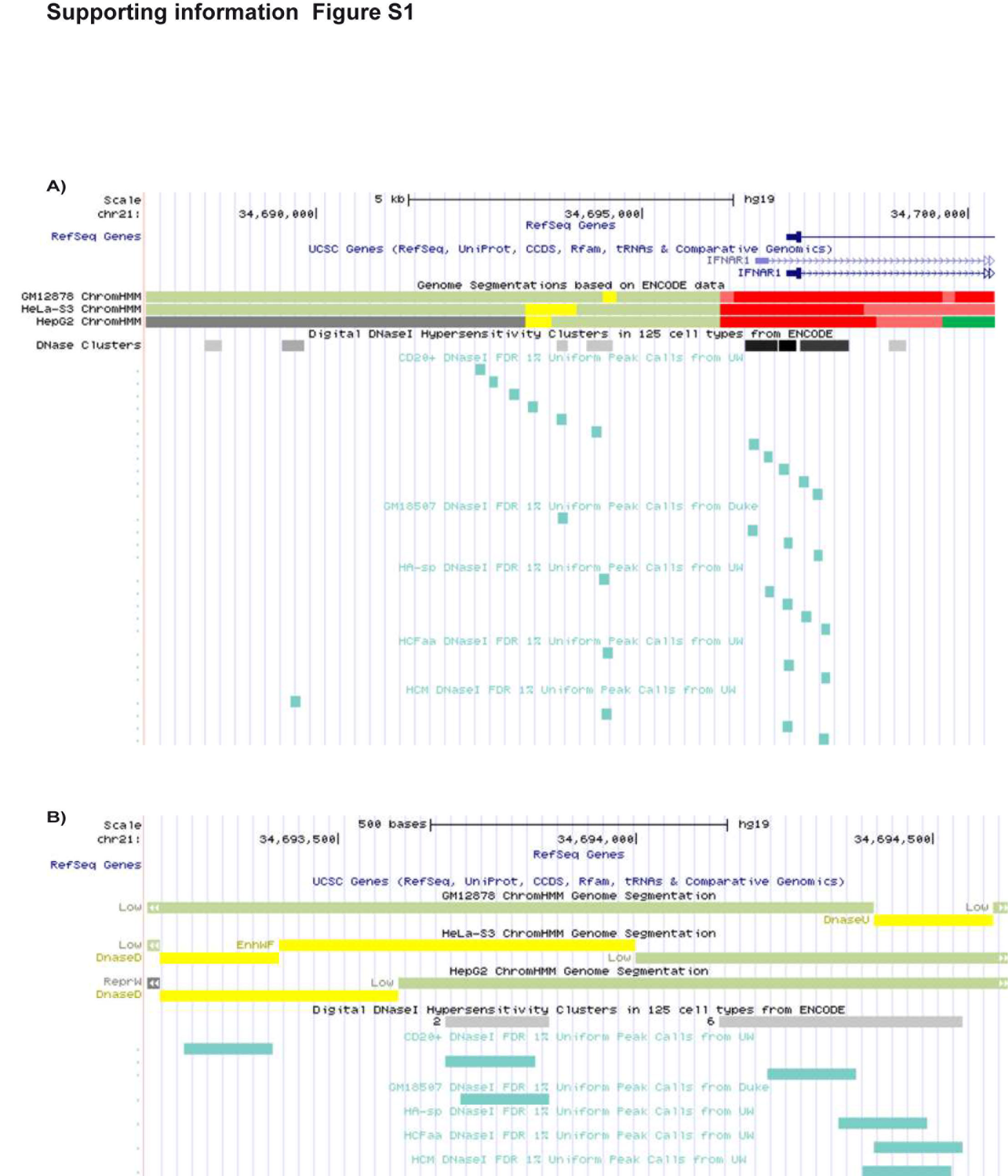

Supplement: Figure S1 — The results from mining of the Encode project dataset ( http://genome.ucsc.edu/ENCODE/ ). Panel A shows ∼13 kb segment centered on HVR with co-ordinates chr21:34687376-34700434 (build 37/hg19) and chr21:33609246-33622304, acc. to build 36/hg18. On the right hand side, this view also includes the promoter/cis-regulatory elements and exon 1 of the IFNAR1 gene. The three colored solid horizontal bars show results (in compressed view) from the ChIP-seq experiments in six cell lines. These were analyzed by a combination of the ChromHMM and Segway programs to perform the segmentations. Three cell lines (GM12878, HeLa-S3 and HepG2) showed “predicted weak enhancer or open chromatin cis regulatory element” in yellow. Other color codes indicate: bright red, predicted promoter region including transcription start site; light red, predicted promoter flanking region; dark green, predicted transcribed region; gray, predicted repressed or low activity region; light green, low activity region. Results from ENCODE data tracks representing the open chromatin signals based on DNase-seq experiments are shown below. Light blue bars indicate DNaseI hypersensitivity clusters in CD20+ cells, GM18507 cells, HA-sp cells, HCFaa cells and HCM cells. Panel B illustrates ENCODE data in greater detail for a segment of 1.45 kb, which corresponds to the region displayed in Fig. 2C (chr21:34693180-34694630, build 37/hg19; and chr21:33615050-33616500, build 36/hg18). The results from ChIP-seq experiments and data tracks representing the open chromatin signals based on DNase-seq experiments are shown in expanded view. The color codes for different predictions are the same as described above for panel A. (TIF) [file pone.0067752.s001.tif]
